# Fangchinoline inhibits the PEDV replication in intestinal epithelial cells via autophagic flux suppression

**DOI:** 10.3389/fmicb.2023.1164851

**Published:** 2023-07-07

**Authors:** Weixiao Zhang, Haiyan Shen, Menglu Wang, Xuelei Fan, Songqi Wang, Nile Wuri, Bin Zhang, Haiyan He, Chunhong Zhang, Zhicheng Liu, Ming Liao, Jianfeng Zhang, Yugu Li, Jianmin Zhang

**Affiliations:** ^1^College of Veterinary Medicine, South China Agricultural University, Guangzhou, China; ^2^Key Laboratory of Livestock Disease Prevention of Guangdong Province, Scientific Observation and Experiment Station of Veterinary Drugs and Diagnostic Techniques of Guangdong Province, Ministry of Agriculture and Rural Affairs, Institute of Animal Health, Guangdong Academy of Agricultural Sciences, Guangzhou, China; ^3^Maoming Branch Center of Guangdong Laboratory for Lingnan Modern Agricultural Science and Technology, Guangzhou, China; ^4^College of Veterinary Medicine, Inner Mongolia Agricultural University, Hohhot, China

**Keywords:** porcine epidemic diarrhea virus, fangchinoline, antiviral agent, autophagy, replication

## Abstract

Animal and human health are severely threatened by coronaviruses. The enteropathogenic coronavirus, porcine epidemic diarrhea virus (PEDV), is highly contagious, leading to porcine epidemic diarrhea (PED), which causes large economic losses in the world's swine industry. Piglets are not protected from emerging PEDV variants; therefore, new antiviral measures for PED control are urgently required. Herein, the anti-PEDV effects and potential mechanisms of fangchinoline (Fan) were investigated. Fan dose-dependently inhibited a PEDV infection at 24 h post-infection (EC_50_ value = 0.67 μM). We found that Fan mainly affected the PEDV replication phase but also inhibited PEDV at the attachment and internalization stages of the viral life cycle. Mechanistically, Fan blocked the autophagic flux in PEDV-infected cells by regulating the expression of autophagy-related proteins and changing PEDV virus particles. In summary, Fan inhibits PEDV infection by blocking the autophagic flux in cells. Our findings will help develop new strategies to prevent and treat PEDV infection.

## 1. Introduction

The enteropathogenic coronavirus, porcine epidemic diarrhea virus (PEDV), causes porcine epidemic diarrhea (PED) (Belouzard et al., 2012). PED damages the intestine via villus atrophy and shedding in pigs of any age. However, PEDV-infected neonatal piglets develop acute watery diarrhea and vomiting, with almost 100% mortality (Alvarez et al., 2015). PED was first reported in 1978; however, in China in 2010, a new PEDV variant strain emerged. Since then, PED has spread worldwide, causing huge losses to the pig industry (Stevenson et al., 2013; Lowe et al., 2014). Commercial PEDV vaccines are available; however, PEDV still persists, and the emergent variant strains make PEDV control more difficult (Pensaert and de Bouck, 1978; Sun et al., 2016; Yu et al., 2018). Consequently, there is an urgent need to develop drugs to prevent porcine PEDV infection.

For thousands of years, herbal medicines have been used to treat viral illnesses, thus representing potential sources of anti-coronavirus treatment (Wen et al., 2011; Ling, 2020; Yang et al., 2020). Fangchinoline (Fan), a traditional Chinese herb monomera, is one of the major dried root alkaloidal components of radix Stephaniae Tetrandrine S. Moore (Zhu, W. et al., 2019). Fan can regulate autophagy and apoptosis, inactivate the inflammasome, and inhibit glutamate release (Lin et al., 2009; Fan et al., 2017; Tang et al., 2017; Liu et al., 2019). To date, most studies have focused on its anticancer activity. Recently, Fan was demonstrated to inhibit HCoV-OC43 infection via an unknown mechanism (Kim et al., 2019).

Autophagy is involved in multiple pathogen infections. Studies have shown that some viruses can induce host cell autophagy, thereby promoting viral proliferation (Xu et al., 2018; Khabir et al., 2020). PEDV was observed to induce reactive oxygen species (ROS)-dependent endoplasmic reticulum (ER) stress-mediated autophagy to promote viral replication (Sun et al., 2021). Moreover, PEDV induced autophagy to benefit its replication (Guo et al., 2017). PEDV replication was promoted by nsp6-induced autophagy, mainly occurring via the PI3K/Akt/mTOR signaling pathway (Lin et al., 2020). Therefore, considering the association between PEDV and autophagy, it would be useful to find drugs that inhibit PEDV via autophagic disruption.

Herein, we aimed to assess Fan's antiviral activity against PEDV in IPEC-J2 cells (porcine intestinal epithelial cells) and determine Fan's antiviral mechanisms by inhibiting autophagic flux. Our study highlights the potential use of Fan to treat PEDV infection. Fan could be used as the basis for anti-viral drugs to curb PED outbreaks.

## 2. Materials and methods

### 2.1. Virus, cells, and reagents

Dulbecco's Modified Eagle Medium (DMEM; Invitrogen, Carlsbad, CA, United States) containing 10% fetal bovine serum (FBS; Gibco, Grand Island, NY, United States) and 1% penicillin–streptomycin (NCM Biotech, Newport, RI, United States) was used to culture African green monkey kidney cells (Vero). IPEC-J2 cells (provided by Dr. Li Wang, Institute of Animal Science, Guangdong Academy of Agricultural Sciences China) were cultured in DMEM/F-12 (Gibco; Invitrogen, Carlsbad, CA, United States) containing 10% FBS and 1% penicillin–streptomycin. Both cell lines were cultured at 37°C in a humidified atmosphere with 5% CO_2_. The PEDV strain GD/HZ/2016 (GenBank Accession: OP191700.1) was isolated, identified, and stored in our laboratory at the Institute of Animal Health, Guangdong Academy of Agricultural Sciences, Guangzhou, China. The GD/HZ/2016 strain was propagated in Vero cells in DMEM containing trypsin. Fangchinoline (HY-N1372A), wortmannin (SL-2052), chloroquine (HY-17589A), and bafilomycin A1 (HY-100558) were purchased from MedChemExpress (Monmouth Junction, NJ, United States).

### 2.2. Cytotoxicity of Fan toward IPEC-J2 cells

IPEC-J2 cells seeded in a 96-well plate were treated with 0, 2.5, 5, 10, 20, 40, and 80 μM Fan for 48 h. A cell counting kit-8 (CCK-8) (Abcam, China) was then used to test cell viability, following the supplier's guidelines. For each concentration, the percentage of viable cells was determined as follows:


$$\begin{array}{l} \left(\frac{ODt}{ODc}\right)\times \;100\%, \end{array}$$

where OD_t_ and OD_c_ are the absorbance of treated and control cells, respectively. We calculated 50% cell cytotoxicity (CC_50_) from data-generated dose–response curves subjected to non-linear regression analysis.

### 2.3. Time-of-addition assay

PEDV strain GD/HZ/2016 was used to infect IPEC-J2 cells at a multiplicity of infection (MOI) of 0.1 and incubated for 1 h. Drug-containing medium (20 μM of Fan) was added at different time points relative to the 1-h period of cell infection with PEDV (MOI = 0.1). Fan was added to the pre-treatment group (Pre) 1 h before the viral infection. It was added at the start of viral incubation in the co-treatment group (Co) and after virus incubation in the post-treatment group (Po). Fan was added throughout the infection period in the full-duration treatment group (Full). It was not added to the virus control group (VC). Following infection, the inoculum was replaced with fresh medium, and the cells were incubated for another 23 h. Thereafter, supernatants and cells were collected for all groups. Quantitative real-time reverse transcription PCR (qRT-PCR) was used to assess viral RNA levels, and the Median Tissue Culture Infectious Dose (TCID_50_) was used to assess the virus titers (Wang et al., 2016; Lai et al., 2020).

### 2.4. Viral attachment, internalization, and replication assays

#### 2.4.1. Attachment assay

Cells were cooled for 1 h at 4°C and treated with various concentrations of Fan (0, 2.5, 5, 10, and 20 μM) and then infected with PEDV GD/HZ/2016 (MOI = 0.5) at 4°C together with various concentrations of Fan and incubated for 1 h, during which time the viruses would adsorb onto the cell membrane but would not penetrate the cells. The cells were washed with ice-cold phosphate-buffered saline (PBS) (Zhu, Z. et al., 2019) and then cultured in the medium for 24 h at 37°C. To assess Fan's effect on virus attachment, the supernatants were subjected to TCID_50_ analysis, and cell samples were collected for Western blotting and qRT-PCR analyses.

#### 2.4.2. Entry assay

PEDV GD/HZ/2016 (0.5 MOI) was used to infect IPEC-J2 cells at 4°C for 1 h. The cells were then washed thrice with cold PBS. Fan (0, 2.5, 5,10, and 20 μM) was added to each sample and incubated for 1 h at 37°C. The cells were washed thrice using PBS, a fresh medium was added, and then, the cells were incubated for 24 h. Next, the intracellular viral RNA, protein levels, and the supernatant virus titers were determined using qRT-PCR, Western blotting, and TCID_50_, respectively.

#### 2.4.3. Replication assay

PEDV GD/HZ/2016 (0.1 MOI) was used to infect IPEC-J2 cells at 37°C for 1 h and then washed thrice using PBS to remove free virus particles. Then, fresh medium with 20 μM Fan was added to the cells. At 9 and 12 h post-infection, the RNA and protein levels of the PEDV N gene were detected using qRT-PCR and Western blotting, respectively.

#### 2.4.4. Release assay

PEDV GD/HZ/2016 (0.1 MOI) was used to infect IPEC-J2 cells at 37°C for 1 h and then washed thrice using PBS to remove free virus particles. The cells were, then, incubated for 24 h at 37°C in the fresh medium containing different concentrations of Fan. Then, the collected cells or cell supernatants were subjected to qRT-PCR assays to determine the number of viral RNA copies.

### 2.5. Regulating cell autophagy using inhibitors

IPEC-J2 cells were treated with wortmannin (0.1 μM), chloroquine (20 μM), or bafilomycin A1 (0.2 μM; all added directly to the medium) for 1 h and then infected with PEDV (MOI = 0.1) for 1 h at 37°C. Then, three washes with PBS removed the unbound viruses, followed by incubation in DMEM/F12 containing 2% FBS with varying concentrations of wortmannin, chloroquine, or bafilomycin A1. The control group was infected with PEDV only. Collected cells were subjected to qRT-PCR and Western blotting to determine the changes in PEDV N mRNA and protein levels. Virus titers were measured in cell supernatants using the TCID_50_ assay.

### 2.6. qRT-PCR

The TaKaRa MiniBEST Universal RNA Extraction Kit (TaKaRa, China) was used to extract total RNA from differently treated IPEC-J2 cells. The qRT-PCR was then performed using a HiScript^®^ II One Step qRT-PCR SYBR Green Kit (Vazyme, China) and the following primers: PEDV-N-F: GCAAAGACTGAACCCACTAAT, PEDV-N-R: GCCTCTGTTGTTACTTGGAG and β-actin-F: GGACTTCGAGCAGGAGATGG, β-actin-R: AGGAAGGAGGGCTGGAAGAG.

### 2.7. Western blotting assay

The procedure was carried out as described in a previous study (Wang et al., 2020). In brief, 150 μl of lysis buffer (ThermoFisher Scientific, China) was used to lyse the cells. The resultant proteins were separated and then transferred to a polyvinylidene fluoride membrane. The membrane was, then, incubated for 1 h in 5% non-fat milk and incubated for 2 h with the following primary antibodies: anti-PEDV N-protein (Medgene Labs, United States), anti-glyceraldehyde-3-phosphate dehydrogenase (GAPDH; ABclonal, China), anti-microtubule-associated protein 1 light chain 3 alpha isoform IIB (LC3II; NOVUS, United States), and anti-sequestosome 1 (SQSTM1/P62; ABclonal, China), followed by three washes with PBST. The membranes were then incubated with horseradish peroxidase (HRP)-conjugated goat anti-mouse and anti-rabbit IgG (H+L) secondary antibodies (Bioworld, China). Immunoreactive protein bands were visualized using an ECL Kit (Millipore, China).

### 2.8. Immunofluorescence assay

Cells were fixed using 4% paraformaldehyde (Beyotime, China) and permeabilized with PBS containing 0.1% Triton X-100. Then, 1% bovine serum albumin (BBI Life Sciences, China) was used to block the cells for 1 h. Then, a mouse anti-PEDV N IgG antibody was incubated with the cells overnight at 4°C and then with Alexa Fluor™ 594 goat anti-mouse IgG (Invitrogen, China) for 1 h at 37°C. Thereafter, cells were incubated with 4′,6-diamidino-2-phenylindole (DAPI, Beyotime) for 5 min after washing with PBS three times. Then, the cells were examined using a differential fluorescence microscope.

### 2.9. Viral titration

Vero cells grown to 70%−80% confluence in a 96-well plate were infected with 10-fold serial dilutions of PEDV (*n* = 4 replicates) and incubated for 72 h at 37°C, followed by Immunofluorescence assay (IFA) assessment. The virus titers were determined using the Reed–Muench method (expressed as TCID_50_ per milliliter).

### 2.10. Calculation of the EC_50_

For the dose-dependent assay, PEDV strain GD/HZ/2016 was infected with IPEC-J2 cells in 12-well cell culture plates, which were then treated with increasing concentrations of Fan from 0 to 20 μM. qRT-PCR determined the PEDV N mRNA levels, and the inhibition value was calculated using the following formula: $\frac{1-\;mRNA\;\left(Fan\right)}{mRNA\;\left(mock\right)}\times 100\%$. A dose–response curve was established using the inhibitory value and the Fan concentration to calculate the EC_50_ (half maximal effective concentration).

### 2.11. Statistical analysis

The data are shown as the mean ± SD. Graph construction and statistical analyses were performed using GraphPad Prism 8.0, and Image J was used to quantify the immunoreactive protein bands. Statistical analysis was performed using a one-way analysis of variance (ANOVA). A *P*-value of < 0.05 indicated statistical significance.

## 3. Results

### 3.1. Fan protects cells against PEDV infection

**Figure 1:**
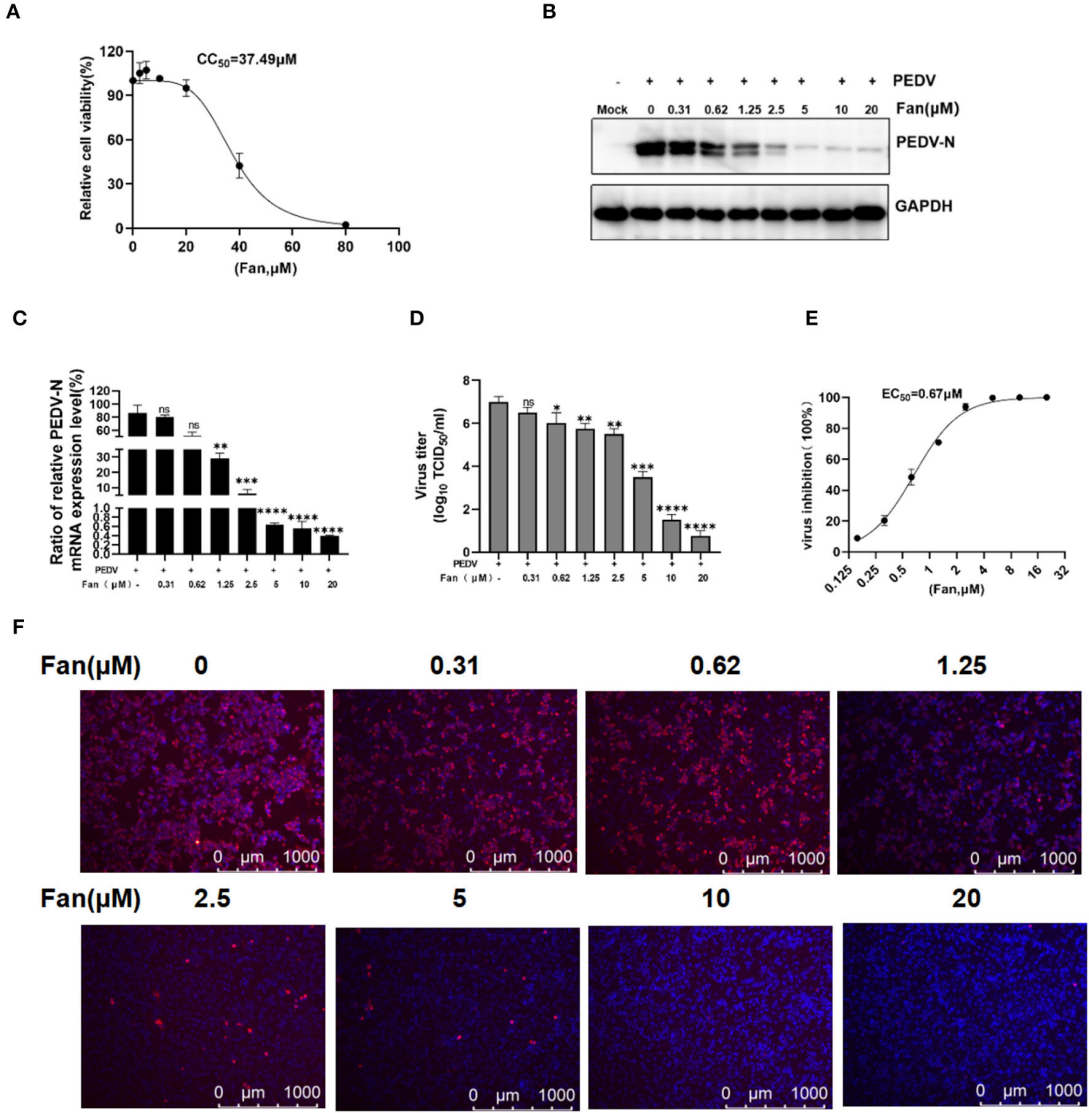
The cellular toxicity and anti-PEDV activity of Fan in IPEC-J2 cell cultures. **(A)** IPEC-J2 cells were treated with various concentrations of Fan at 37°C for 48 h. Cell viability was evaluated using CCK-8 assays. **(B–D)** Fan at various concentrations was used to treat cells for 1 h before PEDV GD/HZ/2016 infection (0.1 MOI), and the cells were then treated with various Fan concentrations for 24 h. At 24 hpi, supernatants and intact cells were collected. **(B)** Western blotting assessment of PEDV N protein levels. **(C)** QRT-PCR quantification of PEDV N mRNA levels. **(D)** TCID_50_ assay to determine the viral titers. **(E)** Assessment of the EC_50_ of Fan toward PEDV infection. **(F)** Effect of Fan on the inhibition of PEDV analyzed using IFA. ^*^P < 0.05; ^**^P < 0.01; ^***^P < 0.001; and ^****^P < 0.0001 indicate significant differences vs. the control group.

We first evaluated Fan-related cytotoxicity toward IPEC-J2 cells using the CCK-8 assay, which revealed that Fan was not cytotoxic to IPEC-J2 cells at 20 μM (CC_50_ = 37.49 μM; Figure 1A). According to the non-cytotoxic range of Fan, we evaluated its inhibition of PEDV infection. The levels of the PEDV N protein declined markedly with increasing Fan concentration (Figure 1B). The qRT-PCR analysis showed that the PEDV N mRNA levels were downregulated by Fan treatment (Figure 1C), with an EC_50_ value of 0.67 μM (Figure 1E). Moreover, 20 μM Fan treatment decreased the virus titers significantly from 10^7^ to 10^0.75^ TCID_50_/ml (Figure 1D). IFA showed that Fan inhibited PEDV infection of IPEC-J2 cells in a concentration-dependent manner (Figure 1F).

### 3.2. Fan-inhibited PEDV in both early and late stages of infection

**Figure 2:**
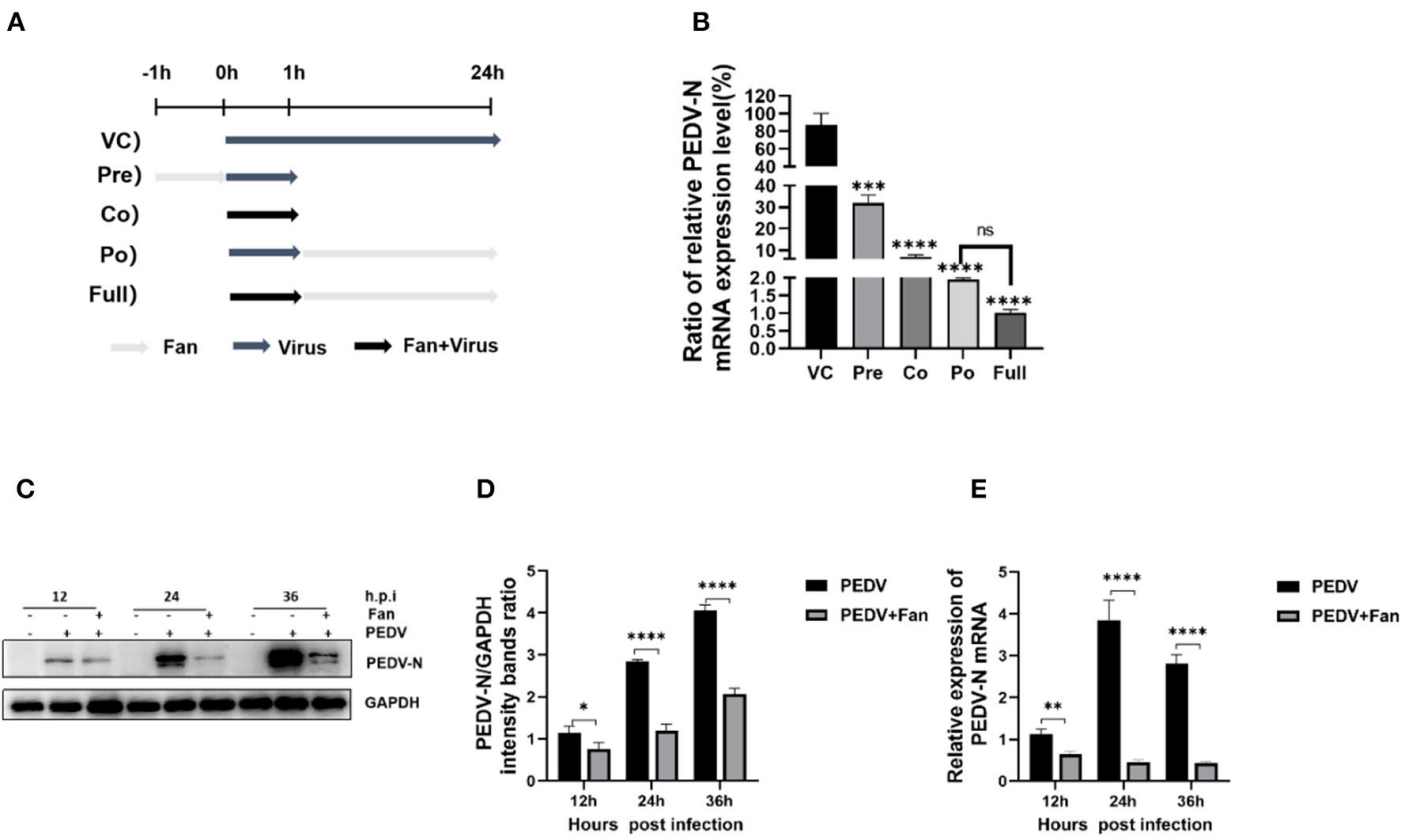
Time of addition assays of Fan. **(A)** IPEC-J2 cells were cultured in 12-well plates and then treated using 20 μM Fan for 1 h before infection with the virus (pre-treatment), for 1 h during infection with the virus (co-treatment), for 23 h after infection with the virus removal (post-treatment), or during the whole infection period (full-time treatment). **(B)** PEDV RNA levels were determined using qRT-PCR analysis. **(C, D)** Western blotting detection of the effects of Fan (20 μM) on PEDV infection in IPEC-J2 cells various times (12–36 h). **(E)** The effects of Fan (20 μM) on PEDV N mRNA in IPEC-J2 cells for various times (12–36 h). VC, virus control; Pre, pre-treatment; Co, co-treatment; Po, post-treatment; Full, full-duration treatment. *P < 0.05; **P < 0.01; ***P < 0.001; and ****P < 0.0001 indicate significant differences vs. the control group.

To determine which stage of PEDV infection was mainly affected by Fan, the time of addition analysis was used (Figure 2A). Fan reduced the PEDV N mRNA levels during the whole viral life cycle. It had the strongest inhibitory effect at the post-treatment stage (~98.86% inhibition), indicating that Fan mainly inhibited PEDV infection at the later stage. Co-treatment suppressed PEDV N RNA levels, suggesting that Fan also affected PEDV infection at the early stage (Figure 2B). Furthermore, the addition of Fan resulted in a significant reduction in PEDV N protein and mRNA levels at 12, 24, and 36 h post-infection (hpi; Figures 2C–E).

### 3.3. Fan inhibits PEDV by affecting viral attachment, internalization, and replication

**Figure 3:**
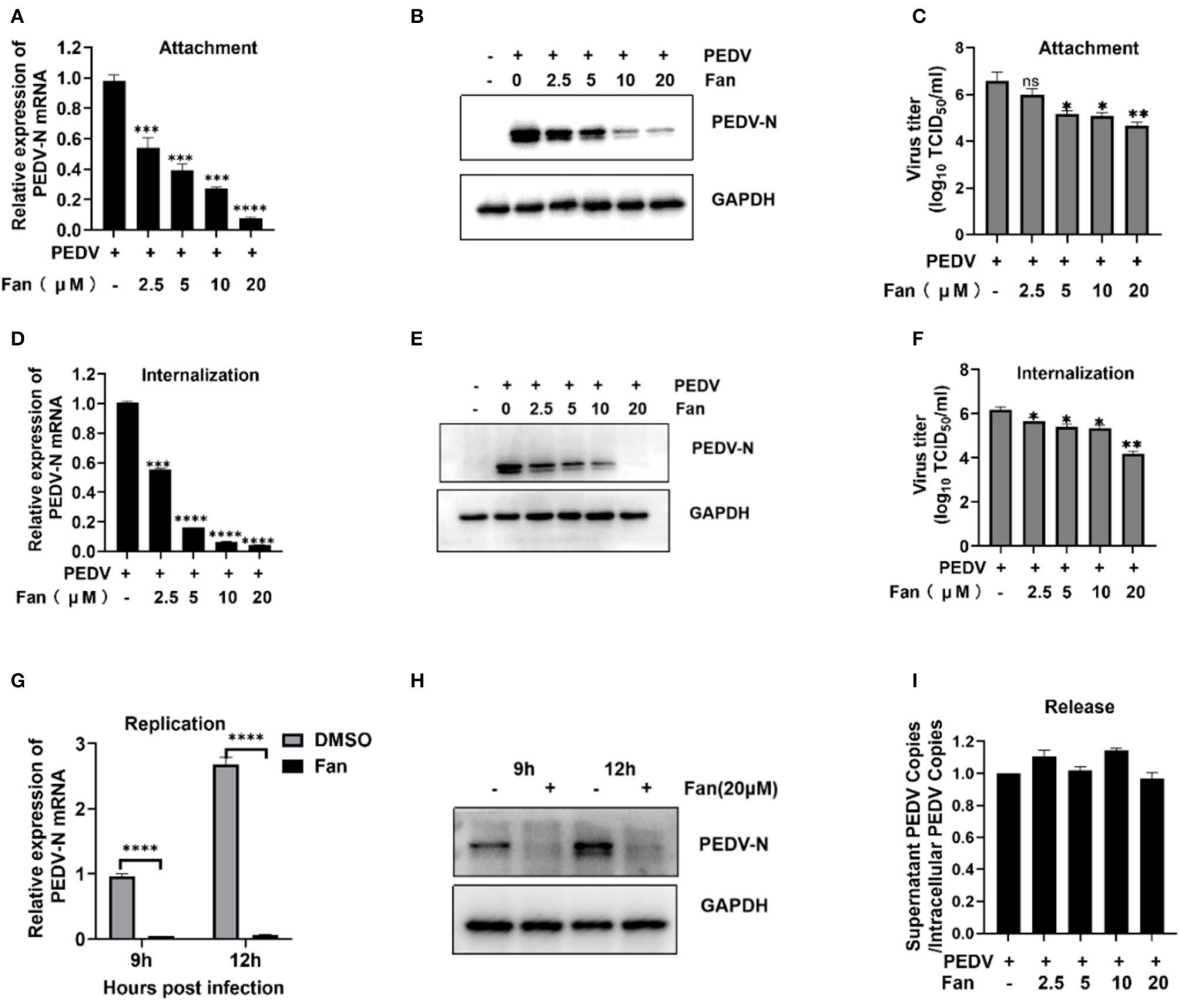
PEDV attachment, internalization, and replication are affected by Fan. **(A–C)** Fan inhibits PEDV by affecting viral attachment, as assessed using qRT-PCR, Western blotting, and TCID_50_ assays. **(D–F)** Fan inhibits PEDV by affecting viral internalization, as assessed using qRT-PCR analysis, Western blotting, and TCID_50_ analysis. **(G, H)** It inhibits PEDV by affecting viral replication as assessed using qRT-PCR analysis and Western blotting. **(I)** At 24 hpi, cellular supernatants and intact cells were obtained, and the PEDV N gene copy number was determined. *P < 0.05; **P < 0.01; ***P < 0.001; and ****P < 0.0001 indicate significant differences vs. the control group.

To further elucidate the inhibition process of Fan at the co-processing stage, binding and entry were performed. The viral attachment tests showed that Fan decreased the levels of PEDV N mRNA (Figure 3A) and protein significantly (Figure 3B), with inhibition rates of 33.77%−91.02%, according to the increasing Fan concentration. Meanwhile, Fan dose-dependently decreased the viral titers, and 20 μM Fan treatment produced a 1.91-log decrease in progeny virus levels (Figure 3C). These results showed that Fan inhibited PEDV attachment to IPEC-J2 cells. In the internalization assay, Fan at 20 μM reduced the PEDV N mRNA levels by 97.63% (Figure 3D), and 20 μM Fan reduced PEDV N protein levels by 97.78% (Figure 3E). PEDV titers decreased by 99.00% in the presence of 20 μM Fan (Figure 3F). To clarify the inhibitory effect of Fan in the later stage of PEDV infection, replication and release were studied. In the viral replication tests, the relative levels of viral RNA and protein treated with 20 μM Fan decreased at 9 and 12 hpi (Figures 3G, H), which suggested that PEDV replication is prevented by Fan treatment. Further experiments showed that Fan treatment did not affect viral release from PEDV-infected cells (Figure 3I).

### 3.4. Fan inhibits autophagic flux in IPEC-J2 cells

**Figure 4:**
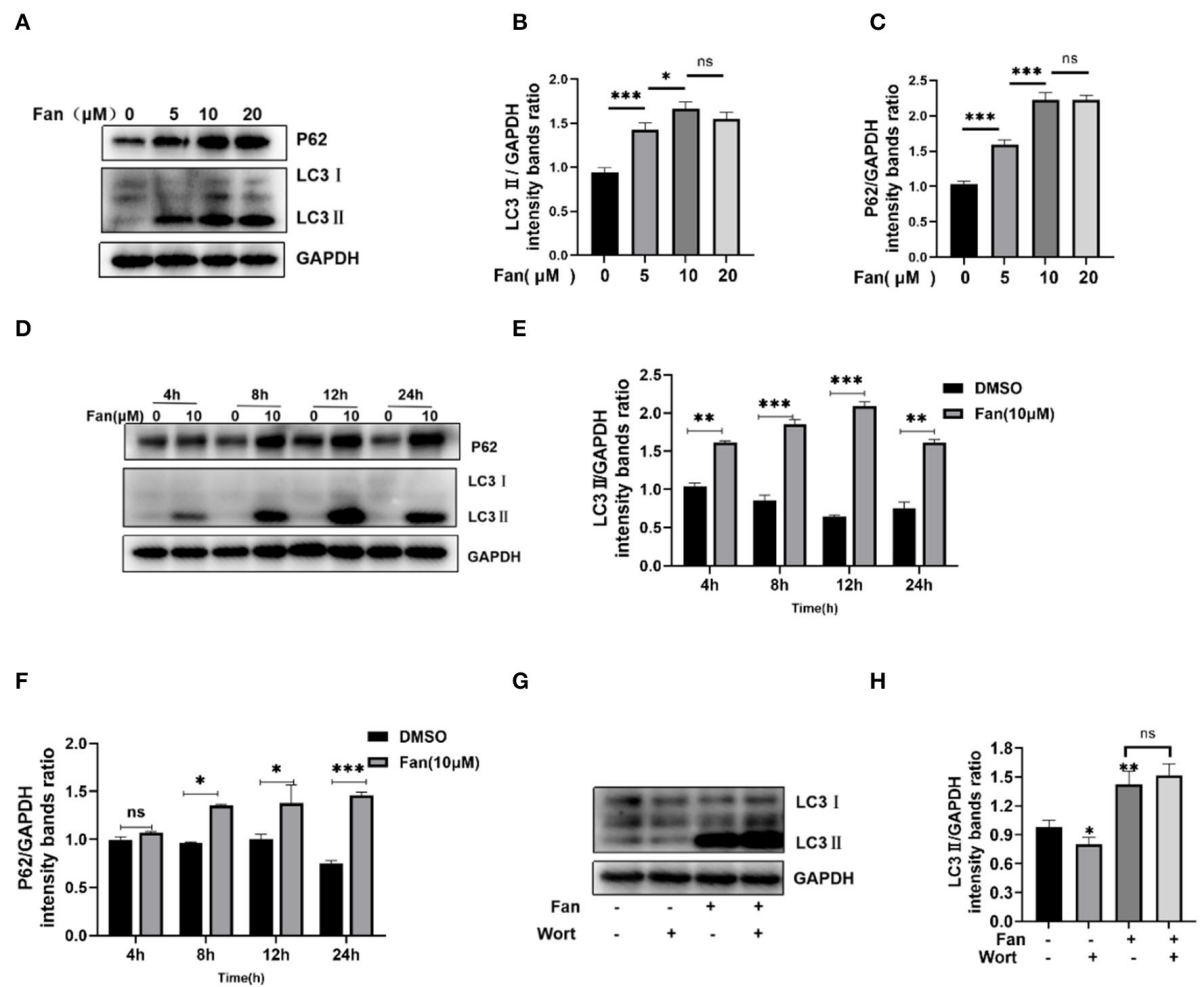
Fan inhibits autophagic flux. **(A–C)** Various concentrations of Fan were used to treat IPEC-J2 cells for 24 h. The cells were then harvested, and Western blotting was used to quantify the levels of autophagy markers LC3II and P62. **(D–F)** Fan at 10 μM was used to treat IPEC-J2 at different times, followed by Western blotting analysis of LC3II and P62. **(G, H)** IPEC-J2 cells were treated with 10 μM Fan, with or without 0.1 μM wortmannin for 24 h. The cells were then harvested, followed by Western blotting analysis of LC3II and P62. Wort, wortmannin. *P < 0.05; **P < 0.01; and ***P < 0.001 indicate significant differences vs. the control group.

Previous research demonstrated that Fan increases LC3II expression (an autophagy marker) by autophagy inhibition (disrupting the fusion of autophagosome and lysosome and lysosome dysfunction) and induction (promoting the nuclear translocation of TFEB), providing insights into the complexity of agent-mediated autophagy (Tang et al., 2017). Autophagosome formation can be monitored by the conversion of LC3I to LC3II. P62/SQSTM1 is a cargo protein receptor that is degraded upon the successful formation of autophagolysosomes (Pal et al., 2014). Therefore, P62 accumulation serves as a marker for autophagy flux inhibition. Our results showed that Fan increased the protein levels of LC3II and P62 in a concentration- and time-dependent manner in IPEC-J2 cells (Figures 4A–F). Increased LC3II and P62 levels are regarded as indicative of defective autophagic flux. Then, we detected the effect of Fan on the early stage of autophagy by adding wortmannin (Figures 4G, H), which showed that wortmannin could not inhibit the Fan-induced increase in LC3II. This indicated that the inhibition of autophagy flux was responsible for the Fan-induced accumulation of autophagosomes.

### 3.5. Inhibition of autophagy downregulates the replication of PEDV in IPEC-J2 cells

**Figure 5:**
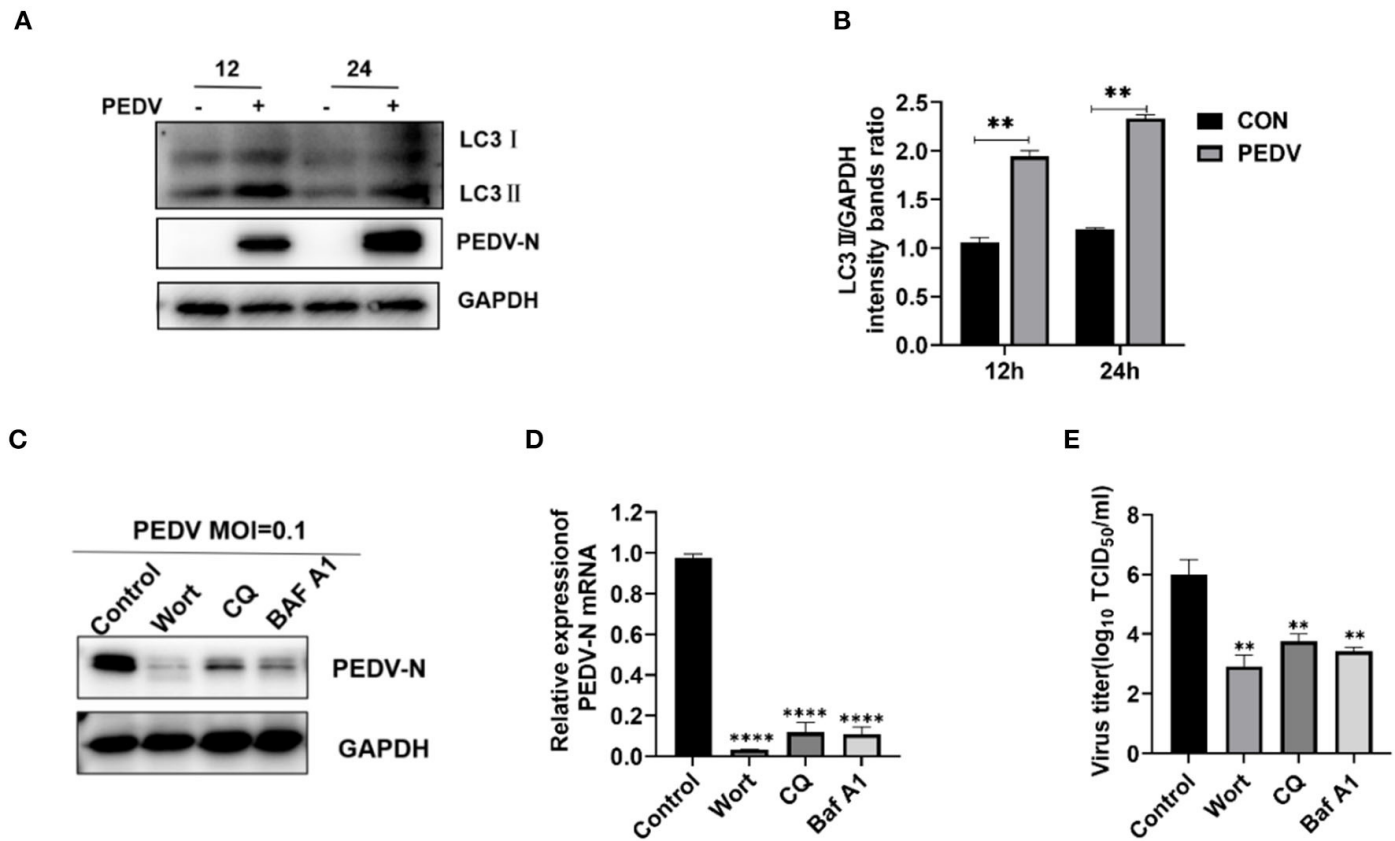
Inhibition of autophagy downregulates the PEDV replication in IPEC-J2 Cells. **(A, B)** At various time points, IPEC-J2 cells were mock infected or infected with PEDV GD/HZ/2016 (MOI = 0.5), followed by Western blotting analysis of autophagy markers. **(C–E)** IPEC-J2 cells were treated with wortmannin (0.1 μM), chloroquine (20 μM), and bafilomycin A1 (0.2 μM) for 1 h and then infected with PEDV (MOI = 0.1). At 24 h hpi, PEDV N mRNA **(C)**, protein **(D)**, and the virus titers **(E)** were assessed using qRT-PCR analysis, Western blotting, and TCID_50_ analysis. Wort, wortmannin; CQ, chloroquine; Baf A1, bafilomycin A1; **P < 0.01, ****, *P* < 0.0001 indicate significant differences vs. the control group.

PEDV infection-induced autophagy positively affects viral replication (Sun et al., 2021; Park et al., 2022). Our results showed that LC3II levels were enhanced with increasing hours post-infection (Figure 5A). The LC3II to GAPDH ratio was significantly higher in PEDV-infected cells than in uninfected cells at 12 and 24 hpi (Figure 5B). To evaluate the effect of autophagy on PEDV infection, IPEC-J2 cells were treated with non-toxic autophagy inhibitors containing either 0.1 μM wortmannin (Supplementary Figure S1A), 20 μM chloroquine (Supplementary Figure S1B), or 0.2 μM bafilomycin A1 (Supplementary Figure S1C). Wortmannin, chloroquine, and bafilomycin A1 treatment decreased the protein and mRNA levels of the N gene in PEDV-infected but not mock-infected cells (Figures 5C, D). At 24 hpi, the PEDV titers in the autophagy inhibitor-treated cells were significantly lower (*P* < 0.05) compared with those in the mock-infected cells (Figure 5E). Thus, the autophagy inhibitors effectively inhibited PEDV infection.

### 3.6. Fan inhibits PEDV replication by inhibiting autophagic degradation

**Figure 6:**
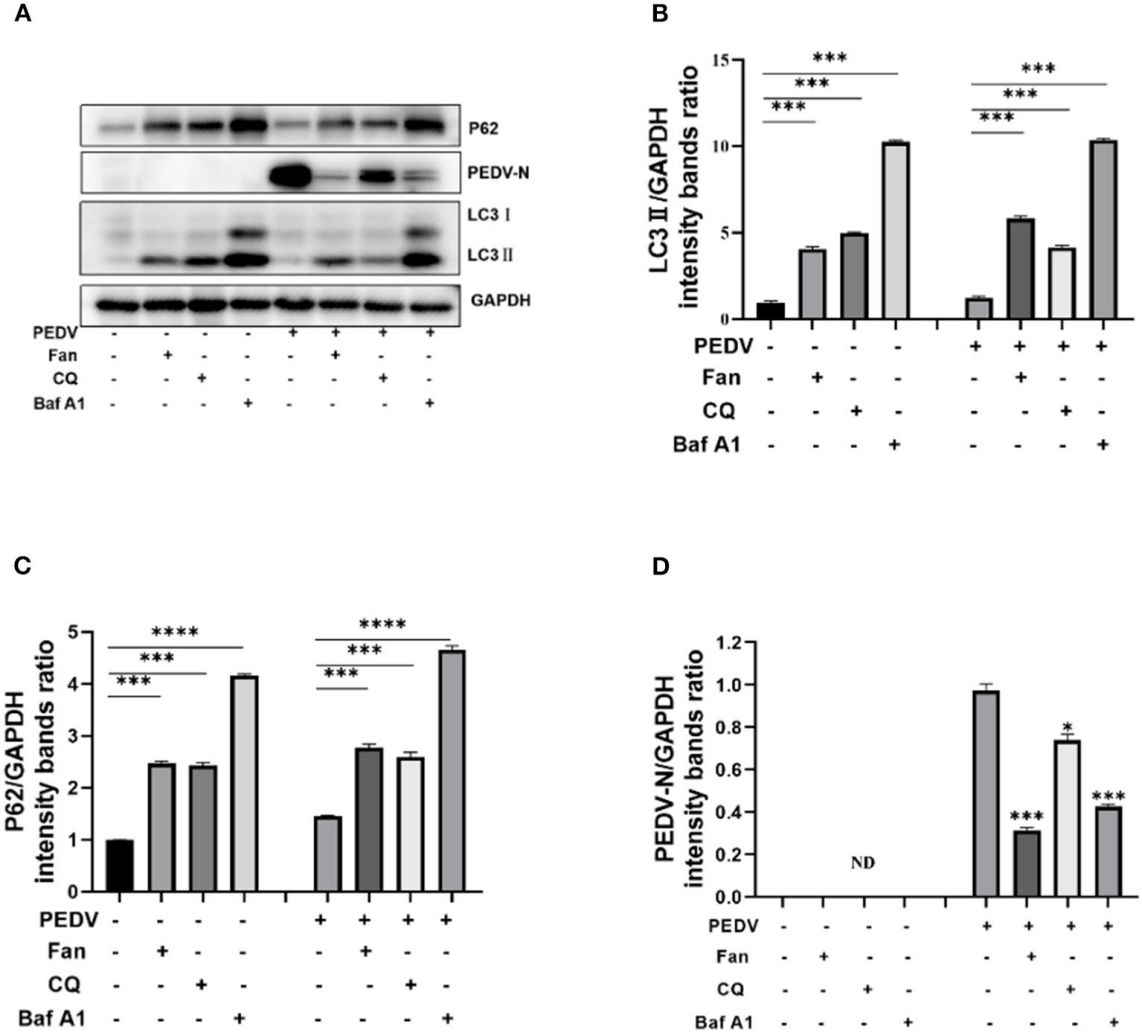
Fan inhibits PEDV replication by inhibiting autophagic degradation. **(A)** IPEC-J2cells were treated with 10 μM fangchinoline, 20 μM chloroquine, and 0.2 μM bafilomycin A1 for 1 h. At 24 h after PEDV (MOI = 0.1) infection, cell lysates were collected for Western blotting as indicated. **(B)** LC3, **(C)** P62, and **(D)** N protein of PEDV were quantitated. CQ, chloroquine; Baf A1, bafilomycin A1. *P < 0.05, ***P < 0.001, and ****P < 0.0001 indicate significant differences vs. the control group.

Taken together, the results suggested that Fan's inhibition of PEDV infection results from the inhibition of autophagy. Consequently, we treated PEDV-infected IPEC-J2 cells with Fan, chloroquine, and bafilomycin A1 and then detected LC3II, P62, and PEDV N protein levels. The results showed increased LC3II and p62 levels in the three drug-treatment groups compared with that in the untreated group. Meanwhile, PEDV N protein levels decreased in the three groups (Figure 6). Thus, Fan inhibits PEDV infection by reducing autophagic flux and inhibiting the formation of autolysosomes.

## 4. Discussion

The recent outbreak of COVID-19 has focused on the research of antiviral medications (Frediansyah et al., 2021; Ohashi et al., 2021; Wu et al., 2022). The enteropathogenic coronavirus PEDV causes a highly contagious enteric infection that results in significant lethality in neonatal piglets (Huang et al., 2013; Shi et al., 2017; Zhang et al., 2019). Therefore, therapeutic strategies to prevent and control PEDV infection are critical.

Fan, a natural bisbenzylisoquinoline alkaloid extracted from *Stephania tetrandra* roots, has numerous pharmacological properties, e.g., anti-inflammatory effects (Choi et al., 2000; Hristova et al., 2003) and inhibition of cancer growth and proliferation (Li et al., 2017; Wang et al., 2017; Zhang et al., 2021; Chen et al., 2022). Although most studies have focused on its anti-cancer activity, its antiviral effect has begun to receive attention. Herein, we found that Fan is a potent natural inhibitor of PEDV infection. Fan dose-dependently restricted PEDV replication in IPEC-J2 cells, with an EC_50_ value of 0.67 μM (Figure 1E). Previous studies have reported the EC_50_ values of Fan against HIV-1 and HCoVs of 0.8–1.7 and 1.01 ± 0.07 μM, respectively (Wan et al., 2012; Kim et al., 2019), which suggests that the effective dose of Fan to inhibit different viruses varies. Fan pre-treatment, co-treatment, and post-infection treatment all exerted an anti-PEDV effect, with post-infection treatment showing the best inhibitory effect. The coronavirus life cycle comprises attachment, internalization, replication, and release, and our results showed that Fan could disrupt multiple steps of PEDV's life cycle, thus inhibiting infection. Fan's inhibitory effect was strongest in the replication stage. A previous study reported that Fan can interfere with the proteolytic processing of gp160 to target Env at the late stage of HIV-1 replication, thereby inhibiting viral propagation (Wan et al., 2012). In addition, it has been reported that some herbal medicines have an antiviral effect on PEDV infection. Li et al. (2020) reported that quercetin could significantly suppress PEDV infection in CCL-81 cells. The possible approach of quercetin for anti-PEDV seemed to inhibit the activity of PEDV 3C-like protease (Li et al., 2020). The other research demonstrated that the aqueous leaf extract of *Moringa oleifera* (MOE) inhibited PEDV replication rather than attachment and internalization. MOE can alleviate oxidative stress and suppress the expression of inflammatory cytokines, which resulted in fewer apoptotic cells during PEDV infection (Cao et al., 2022).

Fan could induce autophagy by activating the AMPK/mTOR/ULK1 signaling pathway in colorectal cancer cell lines (Xiang et al., 2021). Although Fan could increase LC3II levels and the GFP-LC3 puncta formation in non-small cell lung cancer, the use of the autophagy inhibitor bafilomycin A1 did not further increase the Fan-mediated LC3II levels (Tang et al., 2017). This suggested that Fan inhibits autophagic flux. Herein, Fan increased the levels of the autophagy factors LC3II and P62 in IPEC-J2 cells, while the early autophagy inhibitor wortmannin failed to inhibit the increase in Fan-induced LC3II. There has been limited research on the role of autophagy in PEDV replication (Guo et al., 2017; Lin et al., 2020). Given the association between PEDV replication and autophagy and the Fan-mediated regulation of autophagy, we speculate that Fan inhibits PEDV by affecting autophagy. Herein, we demonstrated that PEDV-infected IPEC-J2 cells showed increased conversion of LC3I to LC3II (Figures 5A, B). Accordingly, the replication of PEDV was inhibited by the treatment of IPEC-J2 cells with autophagy inhibitors (wortmannin, chloroquine, or bafilomycin A1). Furthermore, Fan inhibited the late stages of autophagy, resulting in the accumulation of autophagosomes, which has an important function in PEDV infection of IPEC-J2 cells. Similar to chloroquine, Fan inhibits PEDV infection by reducing autophagic degradation in IPEC-J2 cells. Moreover, Fan suppresses PEDV better than chloroquine. Previous research showed that Fan is a potential natural antiviral agent to prevent and treat HCoV-OC43 infection (Kim et al., 2019), which is β-CoV. Therefore, Fan can inhibit α-CoV and β-CoV. Consequently, Fan might have utility as a broad-spectrum anti-coronavirus drug to treat human and animal coronaviruses.

The present study demonstrated that Fan has antiviral activity against PEDV in IPEC-J2 cells via a mechanism involving autophagy regulation. Therefore, Fan might be a promising agent to prevent and treat infection by PEDV or other porcine enteric coronaviruses.

## Data availability statement

The original contributions presented in the study are included in the article/Supplementary material, further inquiries can be directed to the corresponding authors.

## Author contributions

WZ performed the data analysis and drafted the manuscript. WZ, MW, XF, and SW conducted the experiments. NW, BZ, HH, and HS participated in the data analysis. JianmZ and HS conceptualized this study. CZ, ZL, ML, JianfZ, and YL prepared the materials for the experiments. All authors contributed to the article and approved the submitted version.

## Supplementary material

Supplementary Figure S1 Cell viability of IPEC-J2 treated with bafilomycin A1, chloroquine, and wortmannin. IPEC-J2 cells were treated with various concentrations of **(A)** ortmannin, **(B)** chloroquine, **(C)** bafilomycin A1, or **(D)** bafilomycin A1. Then, the cell viability was measured by CCK-8 assay. Wort, wortmannin; CQ, chloroquine; Baf A1, bafilomycin A1.
