# Supplementary figures and images for: Fangchinoline inhibits the PEDV replication in intestinal epithelial cells via autophagic flux suppression
Source: Front Microbiol. 2023 Jul 7;14:1164851. doi: 10.3389/fmicb.2023.1164851 (PMC10360400; doi:10.3389/fmicb.2023.1164851)

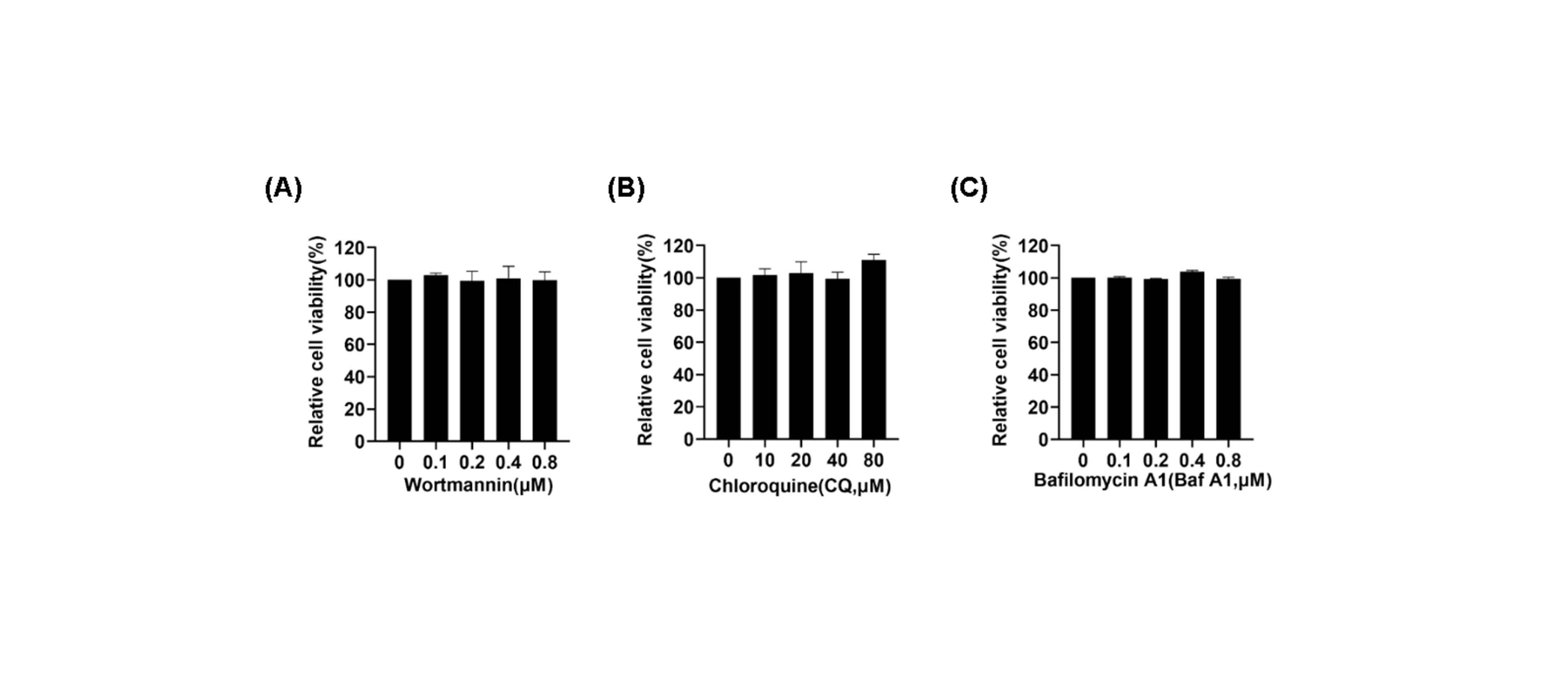

Supplement: Supplementary Figure S1 — Cell viability of IPEC-J2 treated with bafilomycin A1, chloroquine, and wortmannin. IPEC-J2 cells were treated with various concentrations of (A) ortmannin, (B) chloroquine, (C) bafilomycin A1, or (D) bafilomycin A1. Then, the cell viability was measured by CCK-8 assay. Wort, wortmannin; CQ, chloroquine; Baf A1, bafilomycin A1. [file Image_1.TIF]
